# The aftermath of COVID-19: Mortality impact of the pandemic on older persons in Sweden and other Nordic countries, 2020–2023

**DOI:** 10.1177/14034948241253339

**Published:** 2024-06-10

**Authors:** Bo Burström, Örjan Hemström, Megan Doheny, Janne Agerholm, Ann Liljas

**Affiliations:** 1 Department of Global Public Health, Karolinska Institutet, Sweden; 2 Mälardalens University, Västerås, Sweden; 3 Aging Research Centre, Karolinska Institutet, Sweden

**Keywords:** COVID-19, mortality, older people, Nordic countries

## Abstract

**Aims:**

The COVID-19 pandemic hit Sweden harder than the other Nordic countries in the early phase, especially among older persons. We compared the impact of the COVID-19 pandemic on mortality especially among older persons during the period 2020–2022 in Sweden, Denmark, Finland and Norway, using four different outcome measures.

**Methods:**

We compared publicly available information on reported cases and deaths in COVID-19 from the World Health Organization COVID-19 Dashboard, age-specific mortality rates, life expectancy at age 65 years and excess mortality from Nordic Statistics database and national statistics and health agencies in Sweden, Denmark, Finland and Norway.

**Results:**

The pandemic peaked earlier in Sweden than in Denmark, Finland and Norway, where cases and deaths increased more during 2021 and 2022, also reflected in age-specific death rates among persons aged 70+ years. COVID-19 mortality was highest in Sweden, followed by Finland, Denmark and Norway. Life expectancy declined during 2020 in Sweden but more during 2021 and 2022 in Denmark, Finland and Norway. Excess mortality during 2020–2022 was nearly twice as high in Finland as in the other countries.

**Conclusions:**

**COVID-19 mortality was higher in Sweden than in Denmark, Finland and Norway. Life expectancy declined during 2020 in Sweden, was partly regained in 2021 and 2022, while it declined during 2021 and 2022 in Denmark, Norway and Finland. However, excess mortality during 2020–2022 was similar in Sweden, Denmark and Norway and twice as high in Finland. Different mortality outcomes reflect the complexity of the mortality impact of COVID-19.**

## Background

The COVID-19 pandemic hit Sweden harder than the other Nordic countries in the initial phases during spring 2020. Deaths occurred particularly among older persons, where 90% of those who died were aged 70 years or above [1]. Among these, 50% were nursing home residents and 26% received home help [1]. In the neighbouring countries (Finland, Denmark and Norway) overall death rates were lower, and older persons were less affected than they were in Sweden [2,3]. In a comparison of the number of COVID-19 cases and deaths in nursing homes in the United States and European countries until October 2021, the proportion of COVID-19 cases in nursing homes was 21% in Sweden, 10% in Denmark, 2.2% in Finland and 2.3% in Norway [3]. The proportion of COVID-19 deaths in relation to nursing home beds was 7.0% in Sweden, 2.4% in Denmark and 0.7% in both Norway and Finland. COVID-19 deaths in nursing homes constituted 38% of all COVID-19 deaths in Sweden, 37% in Denmark, 32% in Norway and 29% in Finland [3]. Another study comparing COVID-19 incidence in care services for older people during mid-March to April 2020 found that the municipal COVID-19 incidence, cases among staff and lack of testing resources explained most of the variance in the occurrence of COVID-19 cases in residential/home units. Compared with Denmark, the odds ratio for occurrence of COVID-19 among those receiving care services was 1.86 in Sweden, 0.41 in Norway and 0.35 in Finland [4].

The high death toll in COVID-19 among older people in Sweden has been the focus of a government investigation [5] and other studies [6,7]. The lack of preparedness of authorities, slow response of authorities early in the pandemic, the malfunctioning cooperation between health and social care services, the deficiencies in the organization and the resources available to social care and the lack of medical involvement in the care of older persons in long-term care are some factors that have been highlighted [5–7]. Another study investigated whether a larger proportion of vulnerable old people with high mortality risk (‘dry tinder’) in Sweden would explain the higher death toll than in Denmark. However, analyses showed that this could explain only a small proportion of the excess mortality among old people in Sweden, and the authors concluded that policy differences were more important [8].

Moreover, there were large differences between countries in the government response to the COVID-19 pandemic, with Sweden having a less restrictive response than other countries, especially in the early phase [9]. The Oxford Government Response Tracker (OxCGRT) provides a systematic and comparable record of government responses to COVID-19 across 185 countries from 1 January 2020 to 31 December 2022 and covers 23 policy indicators, such as school closures, travel restrictions, mask mandates and vaccination policies [10]. A study comparing the government response in Sweden, Norway, Finland and Denmark using data from the OxCGRT showed a lower response index for Sweden than for the other Nordic countries in March–April 2020. However, over the whole period March–June 2020 the average value of the response index was similar in Sweden, Finland and Norway, although somewhat higher in Denmark. Nevertheless, during March–June 2020 COVID-19 mortality was 10 times higher in Sweden than in Norway and Finland, and five times higher than in Denmark [9]. Although the Nordic countries had a similar approach to COVID-19, Sweden had a less restrictive approach, partly because of a decentralized responsibility for pandemic emergency planning [2]. Denmark had a higher rate of testing than the other countries [11].

During 2021 and 2022, the rates of COVID-19 infection and death in Denmark, Finland and Norway increased more than in Sweden [12], and these countries experienced similar or higher levels of excess mortality [13,14]; a measure that can be used to describe the impact of the COVID-19 pandemic on a population level. Excess mortality is usually presented as a comparison of observed and expected number of deaths in a specific time period (month, year) with another time period (one or several months/years) [14]. The excess deaths in the Nordic countries 2020–2021 were dominated by persons aged 70–89 years [14].

Therefore, it is of interest to study how the COVID-19 pandemic affected mortality in the Nordic countries during the entire time span of 2020–2022.

## Aim

The aim of this study was to compare the impact of the COVID-19 pandemic on mortality, especially among older persons during the period 2020–2022, in Sweden, Denmark, Finland and Norway, using four different outcome measures: COVID-19 mortality, age-specific all-cause mortality, excess mortality and life expectancy at age 65.

## Methods

Data on reported cases and deaths in COVID-19 between 1 March 2020 and 31 December 2023 were obtained from the World Health Organization (WHO) COVID-19 Dashboard [12]. Data on age-specific (in five-year age groups) all-cause mortality rates 2015–2022 among persons aged 70 years and older were obtained from Nordic Statistics [15].

Data on all-cause excess mortality rates in Sweden, Denmark, Finland and Norway were obtained from calculations performed by Statistics Sweden [16]. From the population projections made before the onset of the COVID-19 pandemic, we use projected number of deaths 2020–2022. These are based on long-term trends in mortality rates by sex and one-year age groups and projected changes in the age distribution. All countries have projected declining age-specific mortality by age and sex and an increase in the number of people in old age. A larger number of people in old age is the main reason for the rise in the number of expected deaths over the years 2020 to 2022 even though age-specific mortality is projected to decline over time.

To test whether observed number of deaths (D) was significantly different from the expected number of deaths (E) we assumed this could be the same as testing the significance for standardized mortality ratio with the *Z*-statistic [17]. It is based on the assumption that the expected number of deaths has a Poisson distribution with a standard deviation equal to $\sqrt{E}$ . The calculation is expressed with the following formula [17]:

$$Z=\;\left(D-E\right)/\sqrt{E}$$

*Z*-values greater than 3.29 are significant at the 0.1% level, 2.58<*Z*<3.29 at the 1% level and 1.96<*Z*<2.58 are significant at the 5% level. The test was performed on the annual number of deaths and the total for the three-year period 2020–2022. The expected number of deaths was calculated as an annual mean for a five-year period 2015–2019 and the projected number of expected deaths similarly from population projections in the four Nordic countries.

Life expectancy data at age 65 years was obtained from the Nordic Statistics database [18]. We compared the remaining life expectancy at age 65 years in each country 2005–2022 and the projected trend based on the increase in the 15-year period 2005–2019.

COVID-19 infection rates, case-fatality rates and crude mortality rates were calculated for the countries based on the reported number of cases and deaths, and respective population sizes indicated in the WHO COVID-19 Dashboard [12]. All other data were retrieved directly from the respective public sources.

## Results

### Waves of the COVID-19 pandemic in Sweden, Denmark, Norway and Finland, 2020–2023

**Figure 1.**
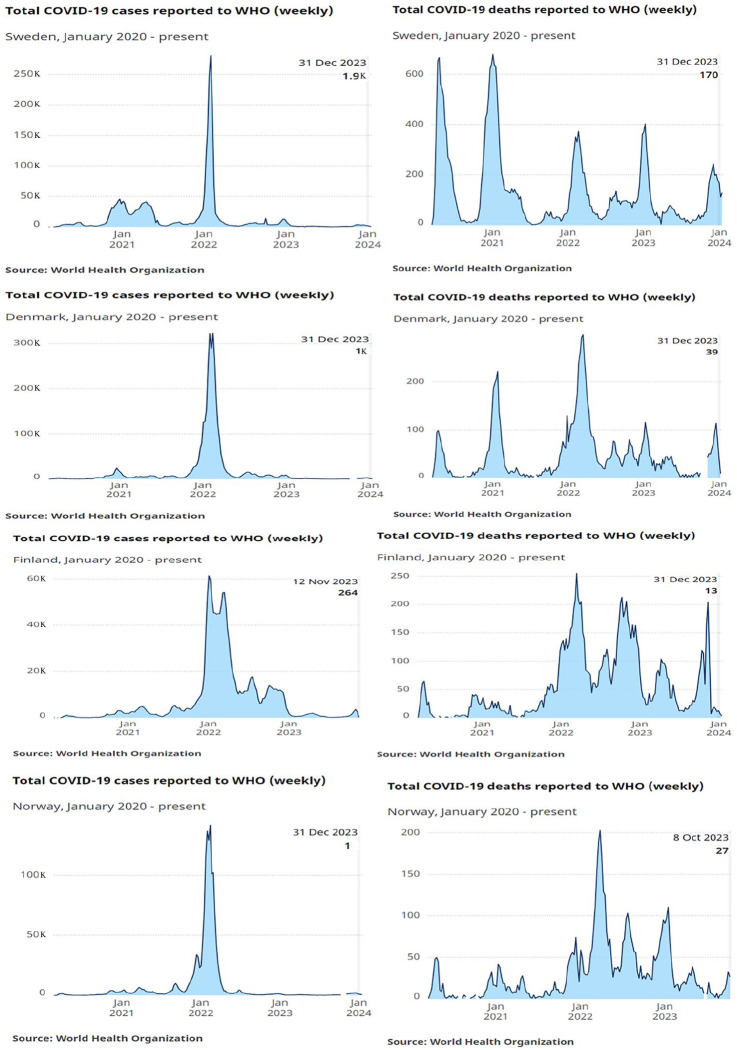
COVID-19 cases and deaths over time, Sweden, Denmark, Finland and Norway. **Source:** WHO COVID-19 Dashboard, accessed 5 February 2024. WHO: World Health Organization

The temporal trends of COVID-19 cases and deaths for Sweden, Denmark, Finland and Norway are shown in Figure 1.

The number of COVID-19 cases and deaths increased rapidly in Sweden from March/April 2020, while the numbers were substantially lower in Denmark, Norway and Finland during 2020. The number of new cases was higher in Sweden compared with Denmark, Finland and Norway at the beginning of 2021. Towards the end of 2021 and early 2022 the number of COVID-19 cases increased in all four countries and remained high in Finland throughout 2022. Vaccines against COVID-19 became available at the beginning of 2021, and different variants of the virus appeared over the period, with differing severity of disease.

#### Sweden

In Sweden, the number of confirmed cases in the first wave of the pandemic in March and April 2020 was quite low, while the number of deaths peaked in the same period. The second wave started in October 2020 with higher number of cases, and a large peak of deaths, continuing into March 2021. The next wave started in October/November 2021, with a higher number of confirmed cases than in the previous periods, but the number had declined to low levels by March/April 2022. The number of daily COVID-19 deaths was increasing during the same period, although the increases were considerably lower than in the previous periods, with a peak in December 2021.

#### Denmark

Denmark had very few confirmed cases of COVID-19 during the first year of the pandemic. Like Sweden, Denmark had one peak of deaths during spring 2020 and one at the end of 2020/beginning of 2021; however, the magnitude was much lower in Denmark than in Sweden. The largest peak of confirmed COVID-19 cases occurred from October 2021 to March 2022, when deaths also peaked. Moreover, the number of daily deaths remained high through December 2022 (Figure 1).

#### Finland

Similarly to Denmark, Finland had a lower number of confirmed COVID-19 cases and deaths The number of cases started to peak in November 2021, declined to a lower level in April 2022 but was elevated through December 2022. The number of daily deaths had two peaks, in March and September 2022, but continued to be high through December 2022 (Figure 1).

#### Norway

As shown in Figure 1, Norway had low numbers of daily confirmed COVID-19 cases and deaths than Sweden during the first wave in 2020. through most of 2020. The number of COVID-19 deaths started to increase during October 2021 and continued to increase throughout the end of 2021. A peak in the number of new cases was seen in December 2021 to March 2022, and deaths continued to be high through December 2022.

### Cumulative number of COVID-19 cases and deaths, case fatality rate and mortality rate per 100,000 population

The cumulative number of COVID-19 cases and deaths from the onset of the pandemic until 31 December 2023 is shown in Table I. The number of confirmed cases was highest in Denmark, followed by Sweden, Norway and Finland, which had lower and more similar rates of infection. The number of COVID-19 deaths was highest in Sweden, followed by Finland, Denmark and Norway. The latest number of deaths from Norway was reported 8 October 2023. The case fatality rate (number of deaths divided by number of cases) was highest in Sweden, followed by Finland, Norway and Denmark. The COVID-19 mortality rate per 100,000 population had a similar distribution (Table I).

**Table I.** Cumulative reported COVID-19 cases and deaths, case fatality rate (deaths/cases), mortality rate per 100,000 population (Denmark, Finland, Norway and Sweden), 3 January 2020 to 31 December 2023.

	Population	Number of cases	Infection rate	Number of deaths	Case fatality rate	Mortality rate per 100,000
Sweden	10,368,969	2,746,618	0.265	26,687	0.0097	257
Denmark	5,825,641	3,432,338	0.589	9426	0.0027	162
Finland	5,529,468	1,499,312	0.271	11,408	0.0076	206
Norway	5,379,839	1,503,875	0.279	5732^a^	0.0038	106

**Source:** WHO COVID-19 dashboard, accessed 5 February 2024.

a Latest report on deaths in Norway 8 October 2023.

WHO: World Health Organization

### Age-specific all-cause mortality rates among persons aged 70+ years, 2015–2022

The COVID-19 pandemic had a differential impact on mortality, hitting older persons hardest.

The age-specific all-cause mortality rates per 100,000 population in five-year age groups from age 70 years, for Denmark, Finland, Norway and Sweden, for the years 2015–2022 are shown in Table II.

**Table II.** Age-specific all-cause mortality rates among persons aged 70 years and older, per 1000 by age group, 2015–2022, Sweden, Denmark, Finland and Norway.

	Age group (years)	2015	2016	2017	2018	2019	2020	2021	2022
**Sweden**	**70–74**	17.5	17.5	16.8	16.7	15.7	16.6	16.0	16.1
	**75–79**	30.0	29.5	29.4	28.9	27.6	30.0	28.2	27.7
	**80–84**	56.1	55.2	54.9	54.6	51.7	56.6	51.3	52.9
	**85–89**	109.0	106.3	108.1	105.8	100.7	111.8	100.7	100.8
	**90–94**	209.9	209.9	210.7	206.6	194.5	218.3	192.6	199.5
	**95+**	415.8	401.0	409.3	404.7	378.6	415.2	373.5	396.4
**Denmark**	**70–74**	21.5	20.7	20.8	20.6	19.9	19.9	20.9	21.0
	**75–79**	35.1	34.7	33.9	34.7	32.9	32.8	33.5	34.6
	**80–84**	65.2	63.4	62.7	63.0	59.5	58.6	59.1	61.0
	**85–89**	114.0	112.8	113.6	116.6	110.2	111.2	115.0	114.5
	**90–94**	200.1	194.8	199.3	204.5	196.3	197.8	203.0	211.2
	**95+**	332.8	331.8	340.2	357.7	340.3	332.5	359.1	366.9
**Finland**	**70–74**	18.2	19.0	18.3	18.1	18.0	18.4	18.8	19.7
	**75–79**	32.0	31.5	30.9	30.5	30.2	28.8	29.6	31.9
	**80–84**	57.2	57.4	56.5	55.5	52.7	55.0	54.8	59.9
	**85–89**	111.1	111.4	106.5	106.3	102.3	101.3	102.4	113.9
	**90–94**	197.2	198.5	194.3	195.9	190.8	187.0	195.1	212.8
	**95+**	354.0	355.1	338.9	354.2	327.4	337.9	346.2	380.2
**Norway**	**70–74**	17.7	17.1	17.1	16.5	16.8	16.6	16.6	17.2
	**75–79**	31.0	29.9	29.7	29.8	28.7	27.2	28.3	30.4
	**80–84**	58.6	56.3	55.6	54.9	53.1	52.0	52.1	54.9
	**85–89**	108.2	106.1	106.0	103.4	100.7	99.9	103.4	109.3
	**90–94**	200.0	193.9	194.3	190.7	188.8	184.4	186.8	207.0
	**95+**	337.8	344.5	340.6	342.8	334.5	329.4	337.3	380.6

**Source:** Nordic Statistics database, latest update 20 December 2023.

In Sweden, mortality rates declined from 2015 until 2019, and increased in all age groups 70 years and older during 2020. However, mortality rates declined during 2021 to similar levels as in 2019. During 2022 the mortality rates among those aged 80 years and below were at similar levels as in 2019, except for an increase in 2022 among those aged 90 years and above.

In Denmark, mortality rates among those aged below 90 years declined from 2015 to 2019, were largely unchanged in all age groups from 2019 to 2020 but increased in all age groups during 2021 and 2022, particularly among those aged 90 years and above.

In Finland, mortality rates declined or were unchanged from 2015 to 2020, with a slight increase during 2021 among those aged 90 years and above. During 2022 mortality rates increased in all age groups 70 years and older in Finland.

In Norway mortality rates declined from 2015 to 2020, with slight increases in 2021 among those aged 85+ years. During 2022, increases in all-cause mortality rates were seen among all age groups 75+ years.

### Excess mortality 2020–2022 compared with 2015–2019

Because of issues concerning classification and attribution of causes of death and varying routines in different countries, many comparative studies have analysed changes in excess all-cause mortality (the difference between observed and expected number of deaths) under the assumption that analyses of trends in this measure would avoid country differences in testing procedures and classification issues in the attribution of cause of death and capture the wider impact of the pandemic [10,11]. If the number of deaths is increased in a given year/period in relation to a comparison year/period, there is excess mortality.

Table III shows data on excess mortality in Denmark, Finland, Norway and Sweden, for separate years and for the period 2020–2022, compared with the mortality level 2015–2019. In 2020 Sweden had by far the highest excess mortality. In 2021, excess mortality was highest in Finland, followed by Denmark, Norway and Sweden. In 2022, excess mortality was highest in Finland, followed by Norway, Denmark and Sweden. During the overall period 2020–2022 Sweden, Norway and Denmark had similar levels of excess mortality while the level was twice as high in Finland (Table III).

**Table III.** Excess mortality in Sweden, Denmark, Finland and Norway 2020–2022, comparison period 2015–2019 and compared with projected number of deaths in population projections. Observed and expected number of deaths, difference in number and per cent.

Country		2020	2021	2022	2020–2022
**Comparison period 2015–2019**					
Denmark	Observed	54,645	57,152	59,435	171,232
	Expected	53,566	53,566	53,566	160,698
	Difference	1079	3586	5869	10,534
	Per cent	2.0***	6.7***	11.0***	6.6***
Finland	Observed	55,488	57,659	63,219	176,366
	Expected	53,723	53,723	53,723	161,168
	Difference	1765	3936	9496	15,198
	Per cent	3.3***	7.3***	17.7***	9.4***
Norway	Observed	40,611	42,002	45,774	128,387
	Expected	40,750	40,750	40,750	122,251
	Difference	–139	1252	5024	6136
	Per cent	–0.3	3.1***	12.3***	5.0***
Sweden	Observed	98,124	91,958	94,737	284,819
	Expected	90,962	90,962	90,962	272,887
	Difference	7162	996	3775	11,932
	Per cent	7.9***	0.9**	3.1***	4.4***
**Comparison population projections**					
Denmark	Observed	54,645	57,152	59,435	171,232
	Expected	54,275	54,728	55,246	164,249
	Difference	370	2424	4189	6983
	Per cent	0.7	4.4***	7.6***	4.3***
Finland	Observed	55,488	57,659	63,219	176,366
	Expected	54,054	54,308	54,589	162,951
	Difference	1434	3351	8630	13,415
	Per cent	2.7***	6.2***	15.8***	8.2***
Norway	Observed	40,611	42,002	45,774	128,387
	Expected	40,835	40,938	41,112	122,885
	Difference	–224	1064	4662	5502
	Per cent	–0.5	2.6***	11.3***	4.5***
Sweden	Observed	98,124	91,958	94,737	284,819
	Expected	90,372	91,104	91,870	273,346
	Difference	7752	854	2867	11,473
	Per cent	8.6***	0.9**	3.1***	4.2***

**p<0.01, ***p<0.001.

Source: National statistical institutes in each country.

### Life expectancy at age 65 years, 2005–2022

**Figure 2.**
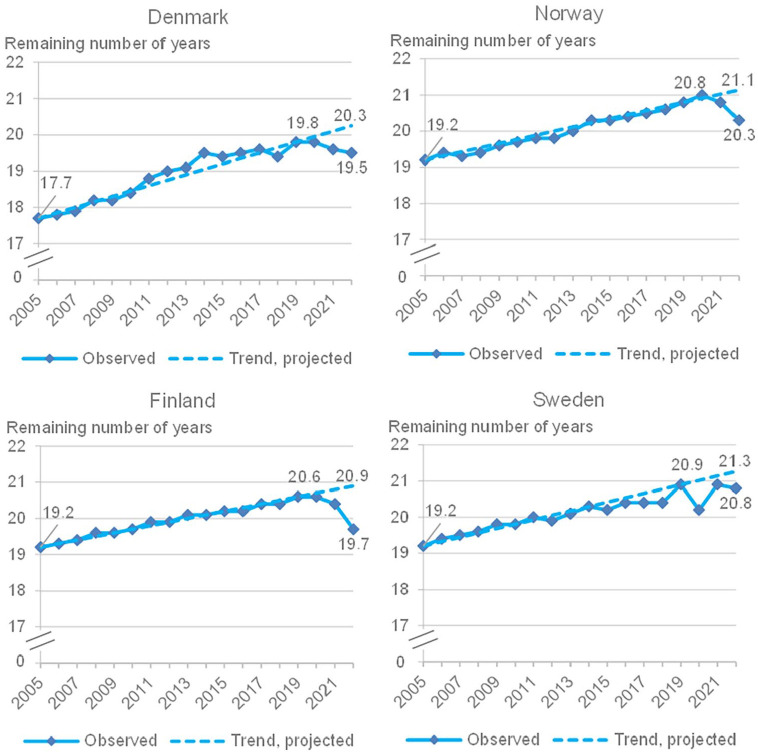
Remaining life expectancy at age 65 years in Denmark, Finland, Norway and Sweden, 2005–2022, and projected trend based on the increase in the 15-year period 2005–2019. **Source:** Nordic Statistics database.

Life expectancy is a standardized measure which is expected to increase over time; if it declines it indicates that mortality increases. Figure 2 shows the remaining life expectancy at age 65 years (total population) in Denmark, Finland, Norway and Sweden, 2005–2022, and the projected trend based on the increase in the 15-year period 2005–2019. Finland, Norway and Sweden all had the same remaining number of years in 2005 (19.2 years), while Denmark had 17.7 years. In 2022 the remaining number of years was 19.5 in Denmark, 19.7 in Finland, 20.3 in Norway and 20.8 in Sweden.

## Discussion

Our comparison illustrates that the mortality impact of the COVID-19 pandemic differs depending on the measure used. This study employs COVID-19 deaths, age-specific all-cause mortality rates among persons aged 70 years and above, excess mortality and life expectancy at age 65 years. The proportion of all COVID-19 deaths occurring among persons 70 years and older was similar and above 80% in all four countries [19,20].

In the early phase of the COVID-19 pandemic during 2020 and into 2021, the number of cases and deaths was considerably higher in Sweden compared with Norway, Denmark and Finland. In Denmark, the number of reported COVID-19 cases peaked at the end of 2021 and early 2022. However, COVID-19 deaths increased also in Denmark during 2020, continuing into 2021 and 2022. In Finland and Norway, the number of COVID-19 cases increased at the end of 2021 and into 2022, and the number of deaths was elevated through 2022.

By the end of 2023, the cumulative COVID-19 infection rates from the onset of the pandemic had become similar in Sweden (0.265), Norway (0.279) and Finland (0.271), while the infection rate was twice as high in Denmark (0.589). The high number of cases in Denmark in late 2021 and the beginning of 2022 was partly due to the spread of the Omicron variant of the COVID-19 virus and the simultaneous lifting of restrictions [21]. However, Denmark also had a much more aggressive testing strategy and during 2021 and through to June 2022 they tested five to seven times more people per 100,000 than the other Nordic countries [11]. The cumulative COVID-19 crude mortality rates per 100,000 population from 2020 to 2023 were 257 in Sweden, 206 in Finland, 162 in Denmark and 106 in Norway. The classification and attribution of cause of death to COVID-19 and the concordance between different sources of data has been discussed [22]. For example, in a comparison of different data sources in Denmark, Friis et al. found that many people died with rather than of COVID-19 [21].

The age-specific all-cause mortality rates among persons 70 years and older 2015–2022 showed an increase in Sweden during 2020 in all five-year classes, with no change or decline during 2020 in Norway, Denmark and Finland. However, in 2021 there were signs of increased rates among the oldest age groups in Denmark, Finland and Norway, which remained or continued to increase during 2022.

More than 80% of COVID-19 deaths in the Nordic countries occurred among persons aged 70+ years [19,20]. A Swedish nationwide population-based study found that frailty and comorbidity were important risk conditions for COVID-19 mortality. Even after controlling for comorbidity, the highest risk for mortality was associated with assisted living, in the home or in nursing home facilities [23]. More knowledge is needed on measures to reduce the impact of COVID-19 on mortality in long-term care. Systematic reviews have been presented on this topic [24,25], indicating that both strategic, tactical and operational measures may be important [25]. Strategic measures include structural characteristics of facilities, tactical include staff issues such as lack of staff and length of work hours while operational issues concern, for instance, use of personal protective equipment and cohort care [25]. However, more research is needed as most studies to date have been based on observational data.

Regarding changes in excess mortality between 2020 and 2022 compared with 2015–2019, the greatest increase was in Sweden during 2020, while excess mortality increased during 2021 and peaked during 2022 in Finland, Norway and Denmark. Between 2020 and 2022, excess mortality was highest in Finland while the other Nordic countries had lower and similar levels of excess mortality. In previous research high excess mortality has been demonstrated to be due to the population structure of the country, particularly advanced age [26,27]. The choice of comparison period may also affect the results. Statistics Sweden used different time periods of comparison: the year 2019, the period 2017–2019 and the period 2015–2019. Using only the comparison year 2019, Sweden had higher excess mortality than the other countries [16], while Sweden had a lower than usual mortality rate that year.

The decline in life expectancy partly reflects the impact of excess mortality, and the timing of the mortality impact of the COVID-19 pandemic. In an analysis of life expectancy changes in 29 countries since 2019 to 2021 [28], Sweden had bounced back in life expectancy from a decline in 2020 to an increase in 2021 (+7.5 months). Norway, Denmark and Finland had no decline in life expectancy in 2020, Norway even had significantly higher life expectancy in 2020 than in 2019. In 2021, Sweden had regained the life expectancy level of 2019. Overall, for the period 2019–2021, Norway had an increase (+1.7 months), Sweden a slight decrease (–0.1 month), Finland a slight decrease (–0.3 month), as well as Denmark (–0.4 month) [28].

Seen over the period 2020–2022, Sweden, Norway and Denmark had similar levels of excess mortality, and excess mortality was highest in Finland. Similarly, the decline in life expectancy at age 65 years occurred during 2020 in Sweden (and partly regained by 2022), while the decline in life expectancy was observed during 2021 and 2022 in Denmark, Norway and Finland.

It appears that part of the higher COVID-19 mortality rate in Sweden could be attributed to the less restrictive response when COVID-19 cases started to occur early in the pandemic compared with in the neighbouring countries [9]. With time, new prevention strategies and treatment regimens became widespread and were quickly implemented in many settings, which improved health outcomes. Features of the overall country responses to the pandemic with different restriction measures in the different countries were also important, as was the roll-out and uptake of COVID-19 vaccination, which greatly reduced and mitigated the mortality impact. In addition, the organization of long-term health and social care of older people, and the implementation of protective measures in the care of older people, is likely to have differed between the Nordic countries. These organizational features should be further studied and compared across the countries to learn from experiences and to better prepare for coming pandemics.

## Conclusions

The COVID-19 pandemic had a greater impact on COVID-19 mortality during 2020–2023 in Sweden than in Denmark, Finland and Norway. Life expectancy at age 65 years declined during 2020 in Sweden, but was partly regained in 2021 and 2022, while life expectancy at age 65 declined during 2021 and 2022 in Denmark, Norway and Finland. However, excess mortality during 2020–2022 was similar in Sweden, Denmark and Norway and twice as high in Finland. Using different mortality outcomes reflects the complexity of the mortality impact of COVID-19.
